# Supplementary material for: Descriptive analysis of interns’ basic psychological needs, burnout and empathy in the COVID-19 pandemic in Ireland
Source: BMJ Open. 2026 Mar 30;16(3):e108611. doi: 10.1136/bmjopen-2025-108611 (PMC13052534; doi:10.1136/bmjopen-2025-108611)
Supplement: online supplemental file 1 [file bmjopen-16-3-s001.zip › bmjopen-2025-108611-20260319144200/suppl_data/Supplementary file.docx]

**Table S1. Spearman correlation coefficients for basic psychological needs, burnout and empathy**

|  | **1**  **EE** | **2**  **PA** | **3**  **DP** | **4**  **AS** | **5**  **AF** | **6**  **CS** | **7**  **CF** | **8**  **RS** | **9**  **RF** | **10**  **Empathy** |
| --- | --- | --- | --- | --- | --- | --- | --- | --- | --- | --- |
| 1. Emotional exhaustion | - |  |  |  |  |  |  |  |  |  |
| 1. Personal accomplishment | -.34** | - |  |  |  |  |  |  |  |  |
| 1. Depersonalisation | .54** | -.23** | - |  |  |  |  |  |  |  |
| 1. Autonomy satisfaction | -.50** | .35** | -.28** | - |  |  |  |  |  |  |
| 1. Autonomy frustration | .66** | -.22 | .46 | -.48** | - |  |  |  |  |  |
| 1. Competence satisfaction | -.33** | .44** | -.30** | .39** | -19** | - |  |  |  |  |
| 1. Competence frustration | .29 | -.32 | .28 | -.18** | .26** | .70**- | - |  |  |  |
| 1. Relatedness satisfaction | -.33** | .36** | -.29** | .53** | -28** | .45** | -.23** | - |  |  |
| 1. Relatedness frustration | .33** | -.31** | .39** | -.30** | -39** | -.36** | .37** | -.57** | - |  |
| 1. Empathy | -.28 | .48 | -.22 | .20** | -.11 | .31** | -.20** | .30** | -.30** | - |
| 1. Burnout | - | - | - | -.35** | .42** | -.32** | .24** | -.26** | .28** | -.21** |

N= 208 cases. **Correlation is significant at 0.01 level (2-tailed). *Correlation is significant at 0.05 level (2-tailed).
