# Supplementary material for: Descriptive analysis of interns’ basic psychological needs, burnout and empathy in the COVID-19 pandemic in Ireland
Source: BMJ Open. 2026 Mar 30;16(3):e108611. doi: 10.1136/bmjopen-2025-108611 (PMC13052534; doi:10.1136/bmjopen-2025-108611)
Supplement: online supplemental file 1 [file bmjopen-16-3-s001.zip › bmjopen-2025-108611-20260319144200/pdf_renditions/bmjopen-2025-108611-S001.pdf]

Table S1. Spearman correlation coefficients for basic psychological needs, burnout and empathy

	1<br>EE	2<br>PA	3<br>DP	4<br>AS	5<br>AF	6<br>CS	7<br>CF	8<br>RS	9<br>RF	10<br>Empathy
1. Emotional exhaustion	-									
2. Personal accomplishment	-.34**	-								
3. Depersonalisation	.54**	-.23**	-							
4. Autonomy satisfaction	-.50**	.35**	-.28**	-						
5. Autonomy frustration	.66**	-.22	.46	-.48**	-					
6. Competence satisfaction	-.33**	.44**	-.30**	.39**	-19**	-				
7. Competence frustration	.29	-.32	.28	-.18**	.26**	.70**-	-			
8. Relatedness satisfaction	-.33**	.36**	-.29**	.53**	-28**	.45**	-.23**	-		
9. Relatedness frustration	.33**	-.31**	.39**	-.30**	-39**	-.36**	.37**	-.57**	-	
10.Empathy	-.28	.48	-.22	.20**	-.11	.31**	-.20**	.30**	-.30**	-
11.Burnout	-	-	-	-.35**	.42**	-.32**	.24**	-.26**	.28**	-.21**

N= 208 cases. **Correlation is significant at 0.01 level (2-tailed). *Correlation is significant at 0.05 level (2-tailed).
