# Supplementary material for: Descriptive analysis of interns’ basic psychological needs, burnout and empathy in the COVID-19 pandemic in Ireland
Source: BMJ Open. 2026 Mar 30;16(3):e108611. doi: 10.1136/bmjopen-2025-108611 (PMC13052534; doi:10.1136/bmjopen-2025-108611)
Supplement: online supplemental file 1 [file bmjopen-16-3-s001.zip › bmjopen-2025-108611-20260319144200/pdf/bmjopen-2025-108611.pdf]

BMJ Open

Descriptive analysis of Interns' basic psychological needs, burnout and empathy in the COVID-19 pandemic in Ireland

Journal:	*BMJ Open*
Manuscript ID	bmjopen-2025-108611.R1
Article Type:	Original research
Date Submitted by the Author:	26-Nov-2025
Complete List of Authors:	Patterson, Aileen; Trinity College Dublin School of Medicine, Medical Education<br>Brady, Conan; Trinity College Dublin<br>Burke, Elaine; Trinity College Dublin School of Medicine, Medical Education<br>Castello, Stefania; Trinity College Dublin<br>Courtney, Laura; Trinity College Dublin School of Medicine, Medical Education<br>F. Curley, Gerard; Royal College of Surgeons in Ireland<br>Donnelly, Suzanne ; University College Dublin School of Medicine<br>Hennessy , Martina ; Trinity College Dublin,<br>McLoughlin, Declan M.; Trinity College Dublin School of Medicine, Psychiatry<br>O'Connell , Finbarr ; Trinity College Dublin School of Medicine, Medical Education<br>Roberts, TE; University of Leeds Faculty of Medicine and Health<br>ten Cate, Olle; Utrecht University, Center for Research and Development of Education<br>Wald, Hedy; Brown University, Warren Alpert Medical School<br>Zgaga, Lina ; Trinity College Dublin, School of Medicine, Public Health and Primary Care
**Primary Subject Heading**:	Medical education and training
Secondary Subject Heading:	Medical management
Keywords:	MEDICAL EDUCATION & TRAINING, Stress, Psychological, Burnout, Empathy

SCHOLARONE™
Manuscripts

I, the Submitting Author has the right to grant and does grant on behalf of all authors of the Work (as defined in the below author licence), an exclusive licence and/or a non-exclusive licence for contributions from authors who are: i) UK Crown employees; ii) where BMJ has agreed a CC-BY licence shall apply, and/or iii) in accordance with the terms applicable for US Federal Government officers or employees acting as part of their official duties; on a worldwide, perpetual, irrevocable, royalty-free basis to BMJ Publishing Group Ltd ("BMJ") its licensees and where the relevant Journal is co-owned by BMJ to the co-owners of the Journal, to publish the Work in this journal and any other BMJ products and to exploit all rights, as set out in our licence.

The Submitting Author accepts and understands that any supply made under these terms is made by BMJ to the Submitting Author unless you are acting as an employee on behalf of your employer or a postgraduate student of an affiliated institution which is paying any applicable article publishing charge ("APC") for Open Access articles. Where the Submitting Author wishes to make the Work available on an Open Access basis (and intends to pay the relevant APC), the terms of reuse of such Open Access shall be governed by a Creative Commons licence – details of these licences and which Creative Commons licence will apply to this Work are set out in our licence referred to above.

Other than as permitted in any relevant BMJ Author's Self Archiving Policies, I confirm this Work has not been accepted for publication elsewhere, is not being considered for publication elsewhere and does not duplicate material already published. I confirm all authors consent to publication of this Work and authorise the granting of this licence.

For peer review only

Descriptive analysis of Interns' basic psychological needs, burnout and empathy in the COVID-19 pandemic in Ireland

Aileen Patterson, Laura Courtney, Olle ten Cate, Trudie Roberts, Hedy Wald, Lina Zgaga, Stefania Castello, Declan M. McLoughlin, Conan Brady, Elaine Burke, Ger Curley, Suzanne Donnelly, Finbarr O'Connell, Martina Hennessy

ABSTRACT

Background The transition from medical student to doctor is recognised as challenging. Interns beginning their first clinical practice during the COVID-19 pandemic faced unprecedented uncertainty with unknown potential consequences for psychological wellbeing and the ability to practice empathetically.

Objectives This study investigated the effect of beginning the practice of medicine during the pandemic on the psychological needs, burnout and empathy of intern doctors.

Design A mixed-methods, sequential cross-sectional study design.

Participants and Setting The National Cohort of intern doctors registered to practice in Ireland in between July 2020 and June 2021 across six Intern Training Networks were invited to participate in an online survey and semi-structure interviews.

Results Interns reported slightly lower or comparable rates of burnout and emotional exhaustion compared to pre-pandemic studies. Frustration of interns autonomy was strongly associated with emotional exhaustion. Higher levels of personal accomplishment were described by interns regularly caring for patients with COVID-19, indicating professional reward from clinical responsibility. Interview analysis revealed 5 themes describing how Interns experienced clinical practice; emotional experience, the need for future reflection, resilience strategies, mitigation measures and preservation of empathy. Protective factors against burnout included initial societal support, team cohesiveness and sharing, and the establishment of an overlap period between outgoing and incoming interns. Postponement of reflection and avoidant strategies employed during the crisis are indicators of future needs of this cohort.

Conclusions

Interns beginning in practice during the pandemic experienced an extremely challenging clinical environment. Our work suggests they met these challenges head-on and retained empathy. Organisational, professional, community and societal responses were critical in

supporting them to achieve relatedness and competence satisfaction. Further work is required to better understand autonomy supportive supervision.

Strengths and limitations of this study

- A multi-centre, mixed method design, using validated instruments and interviews is used to analyse and explore associations among psychological needs satisfaction, burnout and empathy.
- The potential for response bias based on the 21% response rate may limit the generalisability of the findings. There may also be the potential for recall bias based on respondents self-reporting on their experience.
- This study reports correlational associations which do not in themselves indicate casual effect.
- Although multiple testing corrections were considered, the nominal significance threshold of $P \leq 0.05$ was retained. As many variables examined are correlated rather than independent, applying simple corrections such as Bonferroni would therefore be overly conservative and increase the likelihood of Type II errors.
- Specific site influences were not examined and individual exposures were variable over time.

Conflict of interest- none to declare

Acknowledgements- we would like to acknowledge all the intern doctors who practiced during the pandemic, and thank them for their service and commitment. Thank you to the intern coordinators who circulated the invitations and facilitated the study.

Funding: IRC/HRB grant number COV19-2020-114

Word count: 5008 words

Keywords medical education, intern/resident, psychological needs, burnout, empathy

Author Contributions All authors have read and approved the manuscript. MH, AP, EB, FO'C, DMcL, CB, GC, SD, OtC, TR and HW were involved in the conception of the study, including the study design and data analysis. MH,AP, LC carried out the data collection. LC, AP carried out the qualitative analysis, LZ and SC carried out the statistical analysis. All authors contributed to the critical review and formulation of the manuscript. AP is the guarantor.

For peer review only

INTRODUCTION

Internship is a key professional transition period for newly graduated doctors[1,2] that typically necessitates the balancing of uncertainty, gaining of competences and professional insights, and managing new clinical responsibilities.[3] Interns' coinciding roles of learner and caregiver[4] are associated with high levels of stress and negative emotions along with opportunities for rapid personal growth and transformative development.[5] At the onset of the COVID-19 pandemic in 2020, doctors entering the profession experienced interruptions to didactic and clinical teaching, with exams and graduations brought forward to accelerate entry into practice to alleviate workforce shortages.[6,7] These disruptions could undermine newly graduated doctors' confidence, while additional uncertainties about the provision of care, the efficacy of treatments, personal susceptibility to infection and disease transmission,[8] unfamiliar team structures and duties risked compounding this effect.[9,10] Simultaneously, the early phase of the pandemic was characterised by professional pride and public support, which may have ameliorated these pressures.[11] Formal mitigation measures were introduced in anticipation of extra pressure on interns, including employment of additional interns, introducing an overlap period with the outgoing intern cohort, and attachment to one main site for 12 months instead of 3-monthly rotations.[12] Unquestionably, interns beginning their professional practice during the pandemic encountered a different landscape compared to their predecessors, where their needs were secondary to the crisis at hand.

Basic Psychological Needs

Medical and healthcare professionals have traditionally viewed healthcare as a vocation, with caring for patients at its core. Intrinsic motivation, where joy and reward are derived from practice, can be enhanced by environmental supports for three basic psychological needs of autonomy, competence, and relatedness – critical for development and wellbeing.[13] These three needs are the basis of Basic Psychological Needs Theory (BPNT), a mini-theory within Self-determination theory (SDT), that posits that everyone requires the satisfaction of these needs to thrive, whereas frustration of these needs may thwart individual growth and cause psychological distress.[14,15] Autonomy is the need to express volition in one's behaviour, to have choice in one's actions in line with the individual's sense of purpose or motivation. Competence describes the feeling of effectiveness and the capability to perform tasks or actions. Relatedness refers to the feeling of being connected within a community, or a sense

For peer review only - http://bmjopen.bmj.com/site/about/guidelines.xhtml

of belongingness. These psychological needs are important as interns socialise into a profession working in crisis mode and where the values, norms and behaviours of the profession are being tested and moulded in real time. Satisfaction or frustration of these basic psychological needs of interns has consequences for their emotional wellbeing,[16] professional identity formation,[17] and ability to practice empathetically.[18] Clinician psychological distress, measured in terms of burnout[19,20] serves as a negative predictor of empathy, patient care outcomes and medical error.[21-23] Given the significance of burnout on the individual, health system and patient care,[24] it is important to understand what factors contribute to and protect against this serious outcome. Studies from education, sports and business[25,26] and more recently from healthcare[27] show that basic psychological need satisfaction is a key factor affecting burnout and wellbeing.

Burnout and the implications for medical practice

Healthcare workers' wellbeing and burnout have been studied worldwide during the pandemic[28] with prolonged heavy workloads and emotional load reported as risk factors for burnout. Burnout, is defined as a professional psychological stress-induced syndrome recognised by the ICD-11[29] and has serious consequences for the individual and the health systems they operate within. It is a multidimensional construct characterised by emotional exhaustion (EE), depersonalisation (DP) and a reduced sense of personal accomplishment (PA).[30] EE represents a state of being emotionally and physically drained, DP refers to a feeling of detachment or cynicism towards patients and others; often as a coping measure to manage an excessive workload.[31] Reduced PA describes feelings of inefficacy within one's work, which may diminish confidence and increase unhappiness.[19] The cynicism typical of the DP dimension of burnout may be associated with a decrease in empathy toward the emotional needs of patients.[32] Greater healthcare provider empathy has been linked to more positive clinical outcomes for patients,[33,34] including greater patient satisfaction, greater patient compliance with treatment, reduced patient emotional distress and fewer medical errors.[35-38]

Given the pre-existing risk for interns to experience burnout and the detrimental effects on the individual, health system and patient, this study was constructed to examine the effects of practicing during the crisis on interns' psychological needs and distress. Specifically, we investigate the satisfaction and frustration of the three basic psychological needs and their relationship to intern reported burnout and empathy. We hypothesised that satisfaction of

For peer review only

interns' basic psychological needs would correlate with lower burnout and higher empathy results. We further hypothesized that interns would experience higher levels of burnout compared to levels reported in previous studies for cohorts practicing in Ireland, in pre-pandemic times.

METHODS

The study employed a mixed-methods, cross-sectional design with a series of validated surveys to collect quantitative data, and structured phone interviews to gather qualitative data on participants' experiences. The study used STROBE cross sectional reporting guidelines.[38] Data was collected between December 2020 and July 2021 from participants across a range of Irish hospitals. All 995 interns, working in Ireland in 2020, were invited to complete the anonymous survey and participate in an interview. The email invitation was circulated by the local intern co-ordinator. An online food voucher was offered in lieu of lunch to acknowledge participants' time. Ethical approval was granted by the National Research Ethics Committee, 20-NREC-COV-081.

Patient and public involvement

Patients or the public were not involved in the design, conduct, reporting or dissemination of our research. Results from the study will be disseminated through the intern training network.

Measures

The study took place on the online platform Qualtrics XM Platform. Participants received the participant information sheet, and directed to the study consent form before commencing the survey. After providing gender information, participants answered five questions about their proximity to COVID-19 throughout their practice, followed by seven questions assessing their experience of the overlap period. This period is defined as the time at the beginning of internship when new interns were guided, supported and to some extent supervised by the outgoing interns. Participants were presented with three psychological scales.

Basic Psychological Need Satisfaction and Frustration Scale – Work Domain (BPNSF scale)

Psychological need satisfaction/frustration was measured using a version of the BPNSF scale adapted for use in workplace settings.[39] The 24-item scale describes workplace experiences

For peer review only - http://bmjopen.bmj.com/site/about/guidelines.xhtml

and assesses perceived satisfaction or frustration in the three basic psychological needs of– autonomy, competence, and relatedness. Participants rate the extent to which they agree or disagree with each statement on a 7-point Likert scale (1 = *strongly disagree* to 7 = *strongly agree*).[40] Analysis was conducted using each individual subscale and on a composite score of total need satisfaction and frustration.[41]

Jefferson Scale of Empathy (JSE)

The JSE measures the perceived value of the role of physician empathy within patient interactions.[42] The 20-item scale assesses the cognitive and affective dimensions of empathy in three ways: Perspective Taking (ten positively worded items), Compassionate Care (eight negatively worded items), and Standing in the Patient's Shoes (two negatively worded items). Participants rate the extent to which they agree or disagree with each statement on a 7-point Likert scale (1 = *strongly disagree* to 7 = *strongly agree*). Analysis was conducted on the total score.

Maslach Burnout Inventory Human Services Survey for Medical Personnel (MBI-HSS)

Burnout syndrome was assessed using the MBI Human Services Survey for Medical Personnel,[19] a specially developed scale intended for use for healthcare professionals. The survey consists of 22 items that assess three aspects of burnout: emotional exhaustion (EE – the feeling of being emotionally drained and worn-out due to work), depersonalisation (DP – the loss of empathy and the development of cynicism towards others) and personal achievement (PA – a sense of competence in one's work).[43] Participants rate each job-related feeling depending on how frequently they encounter it on a 7-point Likert scale (0 = *never* to 6 = *everyday*). Analysis was conducted on each of the three subscales (EE, DP and PA) using established low, moderate, or high score categories for each subscale[30] (Low (EE = 0-16, DP = 0-6, PA ≥ 39), moderate (EE = 17-26, DP = 7-12, PA = 32-38) and high cut-off points (EE ≥ 27, DP ≥ 13, PA = 0-31)). The PA subscale is negatively scored, wherein higher scores indicate lower PA. Previous research has described the "exhaustion + 1" rule[44] as an effective means of identifying burnout, that is, high scores in EE accompanied by either a high DP and/or a low PA score. This criterion is widely used across existing medical burnout literature.[45]

Phone interview

The structured phone interview was part of a related study examining intern professional identify formation. Interns were asked to recount a meaningful event that occurred during your intern year

For peer review only - http://bmjopen.bmj.com/site/about/guidelines.xhtml

and reflect on how this event contributed to your progression to becoming a fully registered doctor and to consider if there are any changes that you wish to see continued or discontinued.

Quantitative Data and Analysis

Statistical analysis was performed using SPSS statistical software, version 27. A descriptive analysis of all questionnaire scores was performed. Associations between variables were examined using Spearman's rank correlation coefficient. Independent samples t-test (for normally distributed data) or Mann-Whitney U test (for non-normally distributed data) were conducted to assess whether gender affected scores from each questionnaire. One-way ANOVA, Mann-Whitney U, Kruskal-Wallis, Chi-square for independence and Fisher's Exact tests were used to assess differences between groups. Multivariate logistic regression was used to investigate predictors of burnout. Model included gender, contact with COVID-19 patients (regularly, intermittently, rarely), testing positive for COVID-19, and relevant experiences during overlap period, specifically has overlap period helped with interpersonal skills, self-management and scholarship, or professionalism. Final covariate included each individual subscale score for the BPNSF scale, one at a time due to strong correlation between subscales. Analyses were conducted using complete-case (listwise deletion) analysis, where cases with missing data on any variable in the model were excluded. Statistical significance was defined as $p < 0.05$.

Qualitative Data and Analysis

Interviews were recorded and transcribed manually. Interview duration ranged from 9 to 30 minutes (mean 17.5 minutes). Data were analysed using Braun et al's[46] coding reliability thematic analysis (TA). For this study, an initial coding frame was developed following data familiarisation (transcription, reading, and initial analysis of a small portion of the data). The coding frame consisted of a list of codes/themes, each with a label/name, information on how to distinguish the code/theme, and data examples.[47] Codes and themes were mainly identified on a semantic level, reflecting the concepts directly communicated by participants, although some themes express deeper, more latent meanings. Two researchers (AP and LC) independently applied the coding frame to the data, identifying material relevant to each code/theme. Some codes/themes were refined and new themes were added through inductive data engagement,[48] minimising the risk of analytic foreclosure.[49] When all the data had been coded, coders then discussed their own assumptions and positionings.[47] Such

For peer review only - http://bmjopen.bmj.com/site/about/guidelines.xhtml

discussions led to the reaching of consensus on the final coding. The level of agreement between coders, using Cohen's kappa, was 0.95.

RESULTS

A total of 208 interns (21%) responded to the online survey, with 79 (38%) identifying as male, 127 (61%) as female, and 2 (1%) as gender fluid (gender fluid responses were excluded from the gender analysis). 107 (51%) were assigned to a medical team, 86 (41%) to surgery and 15 (7%) to other specialities.

Table 1- Study Participant General Characteristics

	N	%
**Gender**		
Male	79	38
Female	127	61
Genderfluid	2	1
**Team assignment**		
Medicine	107	51
Surgery	86	41
Other	15	7

Burnout and Motivation related scores

Overall, 55% of interns reported high emotional exhaustion (EE), 28% high depersonalisation (DP) and 28% a reduced sense of personal accomplishment (PA) respectively. The mean (standard deviation, SD) score for EE was 26.8 (11.4), indicating a high level of EE. The mean score for DP was 9.5 (6.4), representing a moderate level of DP while the mean score for a reduced sense of personal accomplishment was 34.9 (6.6), signifying a moderate level of reduced PA. A total of 27% of the cohort experienced high scores in EE accompanied by either a high DP and/or a low PA score. Overall and gendered mean scores for interns' basic psychological needs satisfaction and frustration, burnout and empathy are shown in Table 2.

For peer review only

Table 2- Overall and gendered mean scores for interns' basic psychological needs satisfaction and frustration, burnout and empathy (N=208)

	**Mean (SD)**	**Males Mean (SD)**	**Females Mean (SD)**
N	208	68	117
Emotional exhaustion (EE)	26.8 (11.4)	25.1 (10.8)	27.4 (11.4)
Depersonalisation (DP)	9.5 (6.4)	9.6 (6.1)	9.4 (6.5)
Personal accomplishment (PA)	34.9 (6.6)	34.8 (7.4)	34.9 (6.1)
Empathy (JSPE)	113.8 (10.5)	112.1 (11.1)	114.7 (10.0)
BPNSF*			
Autonomy satisfaction (AS)	4.2 (1.2)	4.2 (1.3)	4.2 (1.1)
Autonomy frustration (AF)	4.3 (1.3)	4.5 (1.3)	4.2 (1.3)
Competence satisfaction (CS)	5.4 (0.7)	5.5 (0.9)	5.4 (0.9)
Competence frustration (CF)	3.2 (1.4)	3.1 (1.4)	3.3 (1.4)
Relatedness satisfaction (RS)	5.1 (1.1)	4.9 (1.2)	5.2 (1.0)
Relatedness frustration (RF)**	2.9 (1.1)	3.1 (1.2)	2.8 (1.0)
Missing	16	10	6

* where BPNSF = basic psychological needs satisfaction and frustration, **Significant difference found at a $p < 0.05$ level.

Associations of Basic Psychological Need Satisfaction/Frustration and Burnout/ Empathy

Correlational analysis between all the study variables are reported in Supplementary Table S1. Basic psychological need satisfaction showed positive correlations with personal accomplishment and empathy and negative associations with emotional exhaustion and depersonalisation ($p<.05$). In contrast, basic psychological need frustration was positively correlated with emotional exhaustion and depersonalisation, and negatively associated with personal accomplishment and empathy. In addition, positive correlations were found between high levels of empathy and low levels of burnout. Most correlations were statistically significant with autonomy frustration showing the highest levels of correlation with emotional exhaustion.

For peer review only

Clinical environment factors relevant to Intern experience

How Interns experienced the clinical environment may have been affected by the clinical workload, their personal health experience and the educational value they obtained from practicing during these times.

Clinical practice during the COVID-19 pandemic

The vast majority of interns (80%) reported regularly working with COVID-19 patients during their clinical practice. The frequency and number of COVID-19 patients that interns attended are shown in Table 3. Doctors who had regular contact with COVID-19 patients showed a higher level of PA. A Chi-square test for independence indicated a significant association between having contact with Covid-19 patients and the level of PA, p = 0.02. A one-way ANOVA revealed significant differences in DP scores based on the number of COVID-19 patients, F (4, 182) = 2.55, p = 0.04. Post-hoc tests showed that doctors in contact with 11-20 patients scored lower in DP than those who were in contact with 1-10, 21-30 and over 40 patients. No significant differences were found with EE and PA scores, or with any MBI subscale when using the Kruskal-Wallis test. Besides, no significant associations were found between the number of Covid-19 patients and Overall Burnout, or the levels of EE, DP and PA as assessed with Chi-square and Fisher's Exact tests.

Table 3-Intern level of contact and practice with COVID-19 patients

**The nature of my work has involved contact with Covid-19 patients**	
**Value**	**All**<br>**n, (%)**
Regularly	166, (79.8)
Intermittently	31, (14.9)
Rarely	5, (2.4)
Missing	6, (2.9)
**Total**	208
**To date, how many COVID-19 patients have you personally attended to?**	
**Value**	**All**<br>**n, (%)**
0	0
1-10	25, (12)
11-20	37, (17.8)
21-30	33, (15.9)
31-40	28, (13.5)
Over 40	85, (40.9)
Missing	0
**Total**	208

For peer review only

Personal Risk during Covid-19

The personal health experience of interns depicted in Table 4 was examined as possible additional physical and mental stressors for respondents. There were no significant differences found for burnout or motivation related scores based on personal experience of COVID-19 illness.

Table 4 Personal and Clinical Experience of COVID-19

		**n, (%)**
**Did you self-isolate at home for 2 or more weeks because of possible exposure to COVID-19?**	No	121, (58.2)
	Yes	87, (41.8)
**Have you tested positive for COVID-19?**	No	167, (80.3)
	Yes	41, (19.7)
**Interns who tested positive for COVID-19**		**n, (%)**
**How were you affected by COVID-19?**	no symptoms	7, (3.4)
	mild illness	19, (9.1)
	moderate illness	14, (6.7)
	severe illness	1, (0.5)
**How many weeks did you have symptoms of COVID-19 illness?**	one week	15, (7.2)
	2 weeks	10, (4.8)
	3 weeks	5, (2.4)
	4 weeks	2, (1.0)
	4 weeks +	6, (2.9)
	Missing	3
**Do you feel you have now fully recovered?**	No	11, (5.3)
	Yes	29, (13.9)
	Missing	1

Educational value of practicing during the COVID-19 pandemic

Notwithstanding the clinical and personal challenges new Interns faced on entering practice during the COVID-19 pandemic, there were opportunities for immersion in clinical practice and professional growth. [50] Most Interns (90%) experienced disruption to their final year of studies through either reduced clinical placement/clerkship time (25.5%) and/or having graduating exams moved earlier (63.9%). To compensate the entering cohort were given an overlap period with outgoing Interns. The respondents valued this overlap time for developing patient safety and technical skills, with over 80% rating the experience as having helped a lot or a great deal. Approximately 60% perceived the time as beneficial to the development of their clinical judgement, decision making and interpersonal skills. Over 40%

For peer review only - http://bmjopen.bmj.com/site/about/guidelines.xhtml

rated self-management, scholarship and professionalism positively (Figure 1). Interns had mixed experience of their final year, some had their exams brought forward to allow for an accelerated entry to the workforce, others were removed from their hospital placements and prepared for their final exams and some experienced no interruption.

Demographic Differences

Burnout and motivation results were analysed for significant demographic differences including gender, educational disruptions, team allocation, personal experience and clinical experience of the pandemic. A significant difference in scores of relatedness frustration for males and females was found using independent samples t-tests [males (M = 12.42, SD = 4.87) and females (M = 11.03, SD = 4.1), p = .04], and Mann-Whitney U tests [males (Median= 12) and females (Median= 11), p= .04]. In multivariable analyses models, all subscales (in separate models) were statistically significantly associated with burnout: Autonomy satisfaction (OR=0.38, p=6.12*10-6), Autonomy frustration (OR=1.67, p=0.0024), Competence satisfaction (OR=0.41, p=0.000126), Competence frustration (OR=1.42, p=0.0139), Relatedness satisfaction (OR=0.51, p=0.000226) and Relatedness frustration (OR=1.43, p=0.0394). Other covariates (namely, gender, COVID-19 positivity, frequency of dealing with COVID-19 patients and experience during the overlap period were not associated with burnout.

Thematic Analysis

20 interns volunteered to participate in the qualitative arm. Five main themes were identified relating to the psychological experience of the intern group which provide more insight into the quantitative findings. Specifically, emotional experience, future reflection, resilience, mitigating measures and empathy preservation.

Emotional Experience. The emotional experience of practicing during the COVID-19 pandemic was a central recurring theme, with participants referencing negative emotions, including helplessness (P11, P16), stress (P1, P4, P13, P16), frustration (P4, P13, P15) and feeling overwhelmed (P1, P18). A common experience was described by one intern as:

> *"very mentally... uh, straining to think that (…) the team is working really hard, trying to keep people well and getting everyone home, and you're having... a third of your*

For peer review only - http://bmjopen.bmj.com/site/about/guidelines.xhtml

patients passing away (…) there's this feeling of almost helplessness, because you're trying your best, you're doing what you can with the information and the evidence that you have" (P16).

Many participants described how their practice during COVID appeared to take a physical and psychological toll on them:

"I remember one person was telling me they'd lost a good bit of weight, um, without really realising that they weren't eating at work" (P12)

"you're talking to a lot of families (…) it's their grief that you're dealing with, you didn't know them for that long, but, at the same time trying to... deal with that many people's grief at once, and kind of process your own feelings, it definitely... takes a toll." (P17).

Most participants were aware and direct about the emotional effects they experienced; a small number denied any such feelings. However, of these, most spoke of situations that could typically be considered upsetting and alluded to their difficulty while minimizing the experience.

"we invested a lot in- in those patients, especially the COVID ones, because they- they have a very prolonged, em, ICU stay (…) so we (…) build (…) an emotional link (…) I would say (…) losing patients... it's- it's sort of hard for me, but (…) it's part of (…) doctoring, you know, so (…) to be honest, that doesn't really affect me that much emotionally (…) you know, it is what it is. (laughs)" (P10)

Future Reflection. Many participants recognised that these emotional experiences and their effects would need to be processed in the future. They acknowledged that, due to pressurised conditions, they did not have sufficient time or energy to dedicate to processing their emotions:

"…you're so busy, so you don't really think about it. So it's only now that I've finished, that I think about (…) maybe some of the things that I saw or, that I was managing, by myself, that (…) it was actually a really (…) overwhelming and scary experience." (P18)

Participants faced emotional situations and particularly distressing was witnessing and pronouncing many COVID-related deaths. The frequent proximity to what could be, in many

For peer review only - http://bmjopen.bmj.com/site/about/guidelines.xhtml

cases, considered to be preventable deaths, appeared to have a profound impact on participants:

> "*…before I started my intern year (…) I had never seen somebody die (…) obviously, that's part of every intern year you do... See people pass away and you pronounce them (…) But em, when I worked on a COVID team, like our patients just died, like one after the other ...*" (P18).

Some participants seemed to deflect from the severity of the situation by playing down more serious events:

> "*every on call shift we're pronouncing at least one or two deaths (…) a good way is that it no longer bothers me pronouncing patients but... eh when you stop and think about the number of COVID patients you pronounced (…) it's kind of unsettling (laughs)*" (P12)

Several participants discussed resource prioritisation, and disorientating decision-making when acting in the best interest of patients:

> "*they were oftentimes where it was essentially a judgement call by the consultant in terms of whether we wanted to, uh, discharge somebody who was uh relatively well, and was... still within their kind of red zone of when they could become unwell, or keep them in, um in terms of how aggressively we wanted to start weaning people off oxygen (...) So there were (…) a lot of situations where you weren't sure...*" (P16)

Resilience. A range of coping mechanisms were reported, such as team discussion and sharing experiences, the use of deflection or humour, and for some resignation to the situation, to deal with the pressurised conditions

> "*it felt comfortable to... share that with other people (…) we're very good at talking about all the experiences (…) people were very (…) comforting of each other*" (P17)

Some limited their exposure to COVID-related news and media when outside of work to maintain a strict boundary between the two:

> "*…coming home from work where I really didn't want to watch anything on TV that was any way related to anything medical, anything serious, I just wanted light hearted, em… distraction, basically I again switched off a lot of the news, and I didn't really*

For peer review only - http://bmjopen.bmj.com/site/about/guidelines.xhtml

want to be on social media to see stuff, because I found living the reality of working in covid times (…) challenging enough as it was I didn't really want to engage with people outside of medicine who didn't really know how… bad it was." (P1)

A common coping mechanism that repeatedly came up was "*trying to keep yourself in the right frame of mind*" (P17) and keeping "*everything very stable*" (P4).

Mitigating Measures. Measures that sustained interns' wellbeing included the initial societal support, near-peer interactions, and increased intern numbers. Participants described the public's support as being good in the early stages of the pandemic, but waning as the months wore on:

> "*…at the start, it* (societal support) *was better, eh after the first few months, people got tired of the pandemic (…) stuff stopped coming in and uh, wave two and wave three still hit (…) the goodwill ran out faster than the pandemic did, certainly.*" (P19)

Interns valued the pre-emptive measures introduced to support the health service and their ability to function. The value of employing additional interns and creating an overlap period was highlighted, with one intern commenting how they extended the practice in their new role:

> *"I went in one of the days just to meet the new interns and say look, do you want to sit down and go through the computer system and stuff, because they were the things that I found intimidating, and ….and they both said that this- like this is what we would have liked to get at orientation, rather than... mental health talks - don't get me wrong, mental health talks are important. But at the start of the year, it's not what you're worrying about"*

Preservation of Empathy. The final recurrent theme was how interns strived to practice empathetically, with an awareness of how conditions could lead to empathy erosion. This is evident in the description of communication with patients and their families:

> "*it's very challenging to see an upset patient (…) The importance of just taking those 5 minutes and actually talking to this person… Em… Their loved ones aren't able to come in, there are no visitors allowed (…) you may be one of very few people that they would see all day (…) it doesn't even need to be on a medical basis that you'd be talking to*

For peer review only

> *them about, maybe just to ask them about the hurling, or the football, or the rugby or whatever (…) just to (…) have that interaction with them"* (P3).

Some participants reflected on barriers to empathetic practice typical of the traditional doctor-patient relationship, in the form of personal protection equipment (PPE) and constricted bed visit times, which they described as "*desensitising*" (P15) and "*depersonalising*" (P12).

> *"I tried to stay as (…) in touch with the human side of things as possible, but (…) it was very hard when you weren't seeing patients face to face (…) I expect it was very, very lonely for them and isolating during those times (…) people coming in in full PPE, literally trying to minimise as much time as possible with them (…) they really lost out on the (…) more human side of medicine" (P18).*

Other participants expressed concern on how restrictions, and the increasing number of deaths due to COVID may affect empathy:

> *"…when you're stretched really thin you start to... Not lose your empathy, but (…) it's the empathy and the emotional side that gets reduced (…) And... I think in a time like this, it was probably when they needed it the most."* (P17)

In contrast, one interviewee described the essence of practice when treating a retired clinician that led them to appreciate: "*the concept of common humanity and (…) just how... short life is, and- and how we have to take care of each other…*" (P8)

DISCUSSION

Healthcare workers during the pandemic were found to experience substantial burnout rates across healthcare professions,[28] with similar findings reported for interns beginning practice during the crisis.[51,4] We predicted a higher rate of burnout for interns practicing in Ireland in the pandemic than reported by similar groups working in pre-pandemic conditions. Pre-crisis prevalence burnout rates among intern doctors ranged from 37% to 73%.[52,53] Our results show interns, practicing during the COVID-19 crisis, did not report higher levels of burnout as we hypothesised (27%). Some variation is attributed to differing methodologies of calculation of overall burnout.[45] For more accurate comparison with previous studies, we examined subscale scores, which showed, interns working in more extreme conditions experienced

For peer review only - http://bmjopen.bmj.com/site/about/guidelines.xhtml

similar levels of emotional exhaustion and are less depersonalised than previous cohorts at a similar point in their career.[52,53] However, examination of the intern narratives show we should be cautious in our interpretation of this finding. The range and depth of negative emotion experienced, and the physical and psychological effects on interns may have implications for their future wellbeing. Bearing this in mind, we learned from the qualitative analysis that mitigation measures introduced in response to the pandemic created a supportive clinical environment ensuring that EE and reduced PA is comparable, not worse as we hypothesised, to pre-pandemic levels. This, alongside initial societal support, satisfied the relatedness construct of basic psychological needs, leading to conditions where immediate burnout through depersonalisation was not higher for this cohort. Our findings align with a meta-analysis of 19 controlled interventions of more than 1500 physicians to reduce burnout in physicians,[54] where organisational-directed interventions (workload and rostering) were more likely to be effective compared to individual physician-directed interventions (mindfulness, communicational and educational interventions) that had very small effects on decreasing burnout. This is echoed by a review investigating resilience building and maintenance measures during COVID-19, where organisational and social support were found to mitigate against psychological distress.[55,56]

Overall, satisfaction of interns' three basic psychological needs was significantly associated with lower overall burnout and sub-scale scores. As separate constructs, frustration of their psychological needs resulted in the inverse situation, with interns reporting higher burnout and lower empathy scores where needs were not met.[56]

In terms of competence and relatedness domains and their relationship to burnout, we propose that professional reward from clinical responsibility is indicated as a factor in the protection against burnout. Interns who had regular contact with COVID-19 patients report higher PA than those with less contact. Instead of translating to an occupational stress their basic psychological needs were satisfied in terms of their own professional development and relationships. Related to these findings, trainee physicians in Romania[51] and healthcare professionals in Wuhan, China[57] practicing directly with COVID-19 patients experienced lower burnout than those working in non-COVID areas, with respondents attributing this to greater control, awareness of preventative policies and procedures and feeling more valued. Conflicting evidence from studies in Italy,[58] Turkey,[59] Taiwan[60] and Japan[4] show increased

For peer review only - http://bmjopen.bmj.com/site/about/guidelines.xhtml

levels of burnout for frontline practitioners, where very high incidence of disease, lack of preparatory time and/or unfamiliarity with epidemics may have been additional contributory factors to practitioner burnout.

The sharing of experiences with colleagues was found to be important for maintaining intern resilience. Relatedness frustration was found to be significantly higher for males, although there were no gender differences indicated in levels of reported burnout or subscales.

Regarding clinical experience, interns with a case load of 11-20 COVID-19 patients, at one timepoint, reported significantly less DP, perhaps capturing the point where interns felt productive but not overstretched to a point of diminished service or underused to feel ineffective. Interns personal experience of the pandemic through self-isolation or illness was not found to associate with any burnout subscales. This may be indicative of altruistic internal motivators[61] where personal needs are secondary to personal accomplishment and service.

Descriptions of avoidance strategies are concerning. Previous research[62] showed a significant relationship between such approaches and all domains of burnout, with 64% of interns using such techniques twice as likely to develop burnout over time. Our results present a snapshot of intern perception of their psychological needs and distress and potential alleviating factors. The consequence of practicing during the crisis should not be underestimated, as longitudinal studies show an increase[57] and/ or fluctuating levels[63] of reported distress or burnout for healthcare workers across time.[28,57]

Notwithstanding this study's strengths, including a multi-centre design and the use of validated instruments, we must acknowledge several limitations. As with other cross-sectional studies, this study cannot elaborate on the measured constructs over time. The correlational design of the study revealed significant associations but these relationships in themselves do not indicate causal effect. Although multiple testing corrections were considered, the nominal significance threshold of $P \leq 0.05$ was retained. As many variables examined are correlated rather than independent, applying simple corrections such as Bonferroni would therefore be overly conservative and increase the likelihood of Type II errors. There is a potential for response bias and recall bias as respondents self-reported on their level of burnout. Interns who were experiencing burnout may not have completed the survey or contrarily, may have been more likely to complete the survey due to its relevance to them. The response rate of

For peer review only

21% may not be representative of all interns. Despite these limitations, the response rate is similar to previous studies focussed on this cohort. Prospective research is needed to further explore if the satisfaction of the three basic psychological needs predicts lower levels of burnout, and higher empathy and vice versa.

CONCLUSION

Despite troubling levels of burnout in the Intern population, organisational, community and societal responses to the COVID-19 pandemic illustrated, in practice, environmental conditions supportive of their basic psychological needs of competence and relatedness, and emotional wellbeing. For medical educators, the significant association between autonomy and emotional exhaustion warrants further investigation. Medical education has primarily focused on the development of competence for this cohort, more recently emphasis has been placed on developing clinical responsibility through entrustment activities.[64,65] Relatedness, the sense of belonging to the community, is less emphasised in development and assessment frameworks[65]. However, from this study we see how public, interprofessional, team and peer support were key to the maintenance of resilience, wellbeing and preservation of empathy. Post-crisis, societal fatigue may reduce the sense of support healthcare workers encountered initially and while societal opinion cannot be dictated, continuation of alleviating supervisory and support measures should be considered as part of the longer-term solution to creating an appropriate learning and working environment for interns.

REFERENCES

1. Hannon FB. A national medical education needs' assessment of interns and the development of an intern education and training programme. Med Educ. 2000 Apr;34(4):275-84.
2. Brown J, Chapman T, Graham D. Becoming a new doctor: a learning or survival exercise? Med Educ. 2007;41(7):653-60.
3. Teunissen PW, Westerman M. Opportunity or threat: the ambiguity of the consequences of transitions in medical education. Med Educ. 2011;45(1):51-9.

For peer review only - http://bmjopen.bmj.com/site/about/guidelines.xhtml

4. Kannampallil TG, Goss CW, Evanoff BA, Strickland JR, McAlister RP, Duncan J. Exposure to COVID-19 patients increases physician trainee stress and burnout. PLoS One 2020;15(8):e0237301
5. Bogg J, Gibbs T, Bundred P. Training, job demands and mental health of pre-registration house officers. Med Educ. 2001;35(6):590-5.
6. O'Byrne L, Gavin B, McNicholas F. Medical students and COVID-19: the need for pandemic preparedness. J Med Ethics. 2020;46:623-626.
7. Kinnear B, Zhou C, Kinnear B, Carraccio C, Schumacher DJ. Professional Identity Formation During the COVID-19 Pandemic. J Hosp Med. 2021;16(1):44-46.
8. Stetson GV, Kryzhanovskaya IV, Lomen-Hoerth C, Hauer KE. Professional identity formation in disorienting times. Med Educ. 2020;54(8):765-766.
9. Rana T, Hackett C, Quezada T, et al. Medicine and surgery residents' perspectives on the impact of COVID-19 on graduate medical education. Med Educ Online. 2020;25(1):1818439.
10. Pertile D, Gallo G, Barra F, et al. The impact of COVID-19 pandemic on surgical residency programmes in Italy: a nationwide analysis on behalf of the Italian Polyspecialistic Young Surgeons Society (SPIGC). Updates Surg. 2020;72(2):269-280.
11. Wald HS, Ruddy M. Surreal Becomes Real: Ethical Dilemmas Related to the COVID-19 Pandemic and Professional Identity Formation of Health Professionals. J Contin Educ Health Prof. 2021;41(2):124-129..
12. Department of Health, Ireland, National Public Health Emergency Team (NPHET) COVID-19 Subgroup: Workforce Meeting Tuesday 14th April 2020, https://www.gov.ie/pdf/?file=https://assets.gov.ie/77203/7b8e0a98-10a0-47e3-b3da-df6a1bc48c92.pdf#page=null
13. Ryan, R. M., & Deci, E. L. (2017). Self-determination theory. Basic psychological needs in motivation, development, and wellness.
14. Vansteenkiste and Ryan 2013 On psychological growth and vulnerability: Basic psychological need satisfaction and need frustration as a unifying principle
15. Deci, E. L., & Ryan, R. M. (Eds.). (2004). Handbook of self-determination research. University Rochester Press.

For peer review only

16. Neufeld A, Mossière A, Malin G. Basic psychological needs, more than mindfulness and resilience, relate to medical student stress: A case for shifting the focus of wellness curricula. Med Teach. 2020;42(12):1401-1412.
17. Sawatsky AP, O'Brien BC, Hafferty FW. Autonomy and developing physicians: Reimagining supervision using self-determination theory. Med Educ. 2022;56(1):56-63.
18. Cho E, Jeon S. The role of empathy and psychological need satisfaction in pharmacy students' burnout and well-being. BMC Med Educ. 2019;19(1):43.
19. Maslach C, Jackson SE. The measurement of experienced burnout. Journal of Organizational Behavior. 1981;2(2):99–113.
20. Nituica C, Bota OA, Blebea J, Cheng CI, Slotman GJ. Factors influencing resilience and burnout among resident physicians - a National Survey. BMC Med Educ. 2021;21(1):514.
21. Park C, Lee YJ, Hong M, et al. A Multicenter Study Investigating Empathy and Burnout Characteristics in Medical Residents with Various Specialties. J Korean Med Sci. 2016;31(4):590-597.
22. West CP, Dyrbye LN, Shanafelt TD. Physician burnout: contributors, consequences and solutions. J Intern Med. 2018;283(6):516-529.
23. Salyers MP, Bonfils KA, Luther L, et al. The Relationship Between Professional Burnout and Quality and Safety in Healthcare: A Meta-Analysis. J Gen Intern Med. 2017;32(4):475-482.
24. Francis, R. (2013). Report of the Mid Staffordshire NHS Foundation Trust public inquiry: executive summary (Vol. 947). The Stationery Office.
25. Van den Broeck, A., Vansteenkiste, M., De Witte, H., & Lens, W. (2008). Explaining the relationships between job characteristics, burnout, and engagement: The role of basic psychological need satisfaction. Work & stress, 22(3), 277-294.
26. Baard, P. P., Deci, E. L., & Ryan, R. M. (2004). Intrinsic need satisfaction: a motivational basis of performance and well-being in two work settings 1. Journal of applied social psychology, 34(10), 2045-2068.
27. Cho E, Jeon S. The role of empathy and psychological need satisfaction in pharmacy students' burnout and well-being. BMC Med Educ. 2019;19(1):43.
28. Sharifi M, Asadi-Pooya AA, Mousavi-Roknabadi RS. Burnout among Healthcare Providers of COVID-19; a Systematic Review of Epidemiology and Recommendations. Arch Acad Emerg Med. 2020;9(1):e7.

For peer review only - http://bmjopen.bmj.com/site/about/guidelines.xhtml

29. WHO. ICD-11 for mortality and morbidity statistics (version 04/2019). QD85 Burn-out. https://icd.who.int/browse11/l-m/enP/http:// id.who.int/icd/entity/129180281
30. Maslach, C., Jackson, S. E., & Leiter, M. P. (1996). MBI: Maslach burnout inventory. Sunnyvale, CA: CPP, Incorporated.
31. Maslach C. What have we learned about burnout and health?. Psychol Health. 2001;16(5):607-611.
32. Mercer SW, Reynolds WJ. Empathy and quality of care. Br J Gen Pract. 2002;52 Suppl(Suppl):S9-S12.
33. Del Canale S, Louis DZ, Maio V, et al. The relationship between physician empathy and disease complications: an empirical study of primary care physicians and their diabetic patients in Parma, Italy. Acad Med. 2012;87(9):1243-1249.
34. Rakel D, Barrett B, Zhang Z, et al. Perception of empathy in the therapeutic encounter: effects on the common cold. Patient Educ Couns. 2011;85(3):390-397.
35. Wang H, Kline JA, Jackson BE, et al. Association between emergency physician self-reported empathy and patient satisfaction. PLoS One. 2018;13(9):e0204113.
36. Ogle J, Bushnell JA, Caputi P. Empathy is related to clinical competence in medical care. Med Educ. 2013;47(8):824-831.
37. Neumann M, Wirtz M, Bollschweiler E, et al. Determinants and patient-reported long-term outcomes of physician empathy in oncology: a structural equation modelling approach. Patient Educ Couns. 2007;69(1-3):63-75.
38. von Elm E, Altman DG, Egger M, Pocock SJ, Gøtzsche PC, Vandenbroucke JP; STROBE Initiative. The Strengthening the Reporting of Observational Studies in Epidemiology (STROBE) statement: guidelines for reporting observational studies. J Clin Epidemiol. 2008 Apr;61(4):344-9.
39. Chen, B., Vansteenkiste, M., Beyers, W., Boone, L., Deci, E. L., Van der Kaap-Deeder, J., ... & Verstuyf, J. (2015). Basic psychological need satisfaction, need frustration, and need strength across four cultures. Motivation and emotion, 39, 216-236.
40. Deci, E. L., & Ryan, R. M. (2000). The" what" and" why" of goal pursuits: Human needs and the self-determination of behavior. Psychological inquiry, 11(4), 227-268.
41. Neufeld A, Malin G. Exploring the relationship between medical student basic psychological need satisfaction, resilience, and well-being: a quantitative study. BMC Med Educ. 2019;19(1):405

For peer review only

For peer review only - http://bmjopen.bmj.com/site/about/guidelines.xhtml

42. Hojat M, Mangione S, Nasca TJ, et al. The Jefferson scale of empathy: development and preliminary psychometric data. Educ Psychol Meas. 2001;61:349–65.

43. Maslach C, Leiter MP. Early predictors of job burnout and engagement. J Appl Psychol. 2008;93(3):498-512.

44. Schaufeli, W. B., Leiter, M. P., & Maslach, C. (2009). Burnout: 35 years of research and practice. Career development international.

45. Prentice S, Dorstyn D, Benson J, Elliott T. Burnout Levels and Patterns in Postgraduate Medical Trainees: A Systematic Review and Meta-Analysis. Acad Med. 2020;95(9):1444-1454.

46. Braun V, Clarke V, Hayfield N, Terry G. Thematic Analysis. In: Liamputtong P, editor. Handbook of Research Methods in Health Social Sciences. Singapore: Springer Singapore; 2019. p. 843-60

47. Boyatzis, R. E. (1998). Transforming qualitative information: Thematic analysis and code development. sage.

48. Braun, V., & Clarke, V. (2021). One size fits all? What counts as quality practice in (reflexive) thematic analysis?. Qualitative research in psychology, 18(3), 328-352.

49. Connelly, L. M., & Peltzer, J. N. (2016). Underdeveloped themes in qualitative research: Relationship with interviews and analysis. Clinical nurse specialist, 30(1), 52-57. (Connelly & Peltzer, 2016).

50. Irish Medical Council, Professional Competence Guidelines for Medical Practice, 2011.

51. Dimitriu MCT, Pantea-Stoian A, Smaranda AC, et al. Burnout syndrome in Romanian medical residents in time of the COVID-19 pandemic. Med Hypotheses. 2020;144:109972.

52. Hannan E, Breslin N, Doherty E, McGreal M, Moneley D, Offiah G. Burnout and stress amongst interns in Irish hospitals: contributing factors and potential solutions. Ir J Med Sci. 2018;187(2):301-307.

53. O'Connor P, Lydon S, O'Dea A, et al. A longitudinal and multicentre study of burnout and error in Irish junior doctors. Postgrad Med J. 2017;93(1105):660-664.

54. Panagioti M, Panagopoulou E, Bower P, et al. Controlled Interventions to Reduce Burnout in Physicians: A Systematic Review and Meta-analysis. JAMA Intern Med. 2017;177(2):195-205.

For peer review only

55. Rieckert A, Schuit E, Bleijenberg N, et al, How can we build and maintain the resilience of our health care professionals during COVID-19? Recommendations based on a scoping review. BMJ Open 2021;11:e043718.

56. van der Goot WE, Duvivier RJ, Van Yperen NW, et al. Psychological distress among frontline workers during the COVID-19 pandemic: A mixed-methods study. PLoS One. 2021;16(8):e0255510..

57. Wu Y, Wang J, Luo C, et al. A Comparison of Burnout Frequency Among Oncology Physicians and Nurses Working on the Frontline and Usual Wards During the COVID-19 Epidemic in Wuhan, China. J Pain Symptom Manage. 2020;60(1):e60-e65.

58. Barello S, Palamenghi L, Graffigna G. Burnout and somatic symptoms among frontline healthcare professionals at the peak of the Italian COVID-19 pandemic. Psychiatry Res. 2020;290:113129.

59. Sahin, T., Aslaner, H., Eker, O. O., Gokcek, M. B., & Dogan, M. (2020). Effect of COVID-19 pandemic on anxiety and burnout levels in emergency healthcare workers: a questionnaire study.

60. Sung, C. W., Chen, C. H., Fan, C. Y., Su, F. Y., Chang, J. H., Hung, C. C., ... & Lee, T. S. H. (2020). Burnout in medical staffs during a coronavirus disease (COVID-19) pandemic.

61. Leiter, M. (1991). The dream denied: Professional burnout and the constraints of human service organizations. Canadian Psychology/Psychologie Canadienne, 32(4), 547.

62. Roslan NS, Yusoff MSB, Ab Razak A, et al. Training Characteristics, Personal Factors and Coping Strategies Associated with Burnout in Junior Doctors: A Multi-Center Study. Healthcare (Basel). 2021;9(9):1208. Published 2021 Sep 14. doi:10.3390/healthcare9091208.

63. Shanafelt TD, West CP, Sinsky C, et al. Changes in Burnout and Satisfaction With Work-Life Integration in Physicians and the General US Working Population Between 2011 and 2020. Mayo Clin Proc. 2022;97(3):491-506.

64. O'Dowd E, Lydon S, O'Connor P, Boland J, Offiah G, Byrne D. The development of a framework of entrustable professional activities for the intern year in Ireland. BMC Med Educ. 2020;20(1):273.

65. Hennus MP, Nusmeier A, van Heesch GGM, Riedijk MA, Schoenmaker NJ, Soeteman M, Wildschut ED, Fawns T, Ten Cate O. Development of entrustable professional activities

For peer review only

For peer review only - http://bmjopen.bmj.com/site/about/guidelines.xhtml

for paediatric intensive care fellows: A national modified Delphi study. PLoS One. 2021 Mar 18;16(3):e0248565.

66. Sawatsky AP, Rea JR, Hafdahl LT, Vaa Stelling BE, Huber JM, Wingo MT, Leasure EL. From Apprenticeship to Assembly Line: Recovering Relationships in Medical Education. J Grad Med Educ. 2023 Dec;15(6):627-631. doi: 10.4300/JGME-D-23-00468.1. PMID: 38045942; PMCID: PMC10686647.

For peer review only

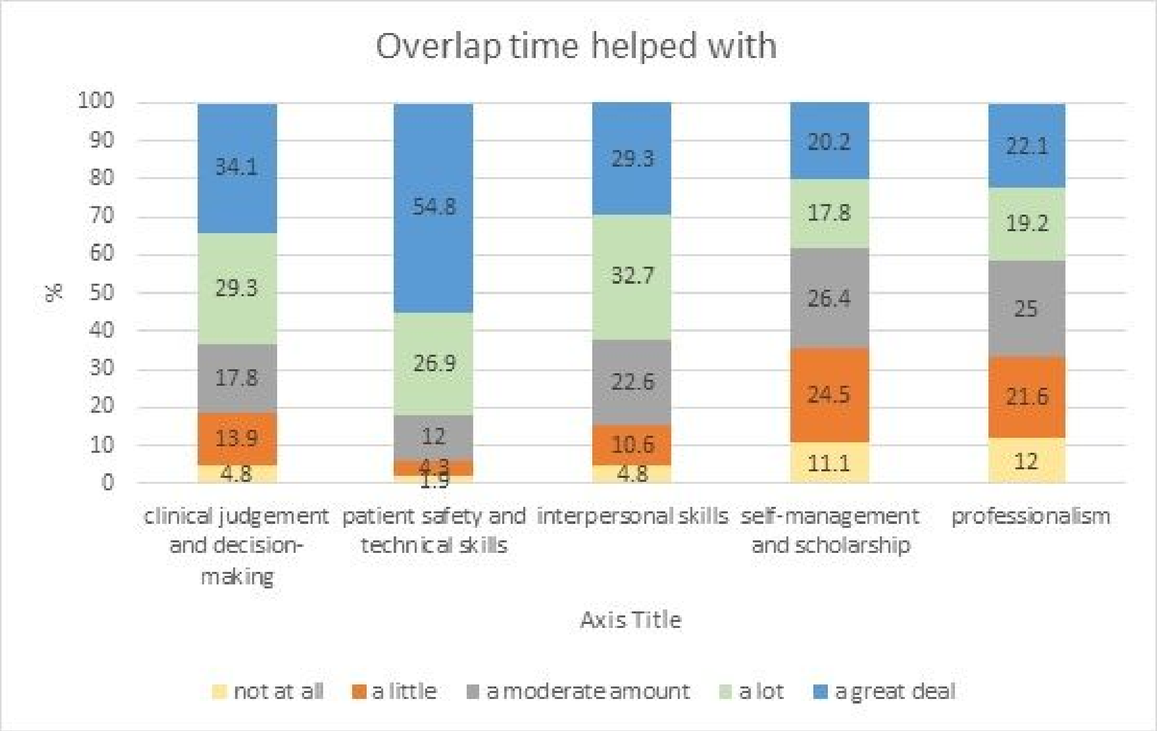

Figure 1- Intern rating value of overlap period for achievement of core domains of practice

For peer review only

Table S1. Spearman correlation coefficients for basic psychological needs, burnout and empathy

	1 EE	2 PA	3 DP	4 AS	5 AF	6 CS	7 CF	8 RS	9 RF	10 Empathy
1. Emotional exhaustion	-									
2. Personal accomplishment	-.34**	-								
3. Depersonalisation	.54**	-.23**	-							
4. Autonomy satisfaction	-.50**	.35**	-.28**	-						
5. Autonomy frustration	.66**	-.22	.46	-.48**	-					
6. Competence satisfaction	-.33**	.44**	-.30**	.39**	-19**	-				
7. Competence frustration	.29	-.32	.28	-.18**	.26**	.70**-	-			
8. Relatedness satisfaction	-.33**	.36**	-.29**	.53**	-28**	.45**	-.23**	-		
9. Relatedness frustration	.33**	-.31**	.39**	-.30**	-39**	-.36**	.37**	-.57**	-	
10.Empathy	-.28	.48	-.22	.20**	-.11	.31**	-.20**	.30**	-.30**	-
11.Burnout	-	-	-	-.35**	.42**	-.32**	.24**	-.26**	.28**	-.21**

N= 208 cases. **Correlation is significant at 0.01 level (2-tailed). *Correlation is significant at 0.05 level (2-tailed).

For peer review only
