# Supplementary material for: Descriptive analysis of interns’ basic psychological needs, burnout and empathy in the COVID-19 pandemic in Ireland
Source: BMJ Open. 2026 Mar 30;16(3):e108611. doi: 10.1136/bmjopen-2025-108611 (PMC13052534; doi:10.1136/bmjopen-2025-108611)
Supplement: online supplemental file 1 [file bmjopen-16-3-s001.zip › bmjopen-2025-108611-20260319144200/bmjopen-2025-108611-manifest.html]

Manifest file for export bmjopen-2025-108611-20260319144200


|  |  |
| --- | --- |
| File name: bmjopen-2025-108611-20260319144200 | |
| Export Date: 19-Mar-2026 | |
| Output Format: XML (ScholarOne DTD) | |
| bmjopen-2025-108611-20260319144200/doc/Main\_document.docx | Version 1.1 |
| bmjopen-2025-108611-20260319144200/pdf\_renditions/bmjopen-2025-108611-File001.pdf | Version 1.1 |
| bmjopen-2025-108611-20260319144200/graphic/figure1.docx | Version 1.0 |
| bmjopen-2025-108611-20260319144200/pdf\_renditions/bmjopen-2025-108611-File002.pdf | Version 1.0 |
| bmjopen-2025-108611-20260319144200/suppl\_data/Supplementary file.docx | Version 1.0 |
| bmjopen-2025-108611-20260319144200/pdf\_renditions/bmjopen-2025-108611-S001.pdf | Version 1.0 |
| bmjopen-2025-108611-20260319144200/doc/bmjopen-2025-108611.PV.pdf | Version 1.0 |
| bmjopen-2025-108611-20260319144200/doc/bmjopen-2025-108611.RH.pdf | Version 1.0 |
| bmjopen-2025-108611-20260319144200/pdf/bmjopen-2025-108611.pdf |  |
| bmjopen-2025-108611-20260319144200/bmjopen-2025-108611-metadata.xml |  |
| bmjopen-2025-108611-20260319144200/s1.dtd |  |
| manifest.html | This document |
