# Supplementary figures and images for: Descriptive analysis of interns’ basic psychological needs, burnout and empathy in the COVID-19 pandemic in Ireland
Source: BMJ Open. 2026 Mar 30;16(3):e108611. doi: 10.1136/bmjopen-2025-108611 (PMC13052534; doi:10.1136/bmjopen-2025-108611)

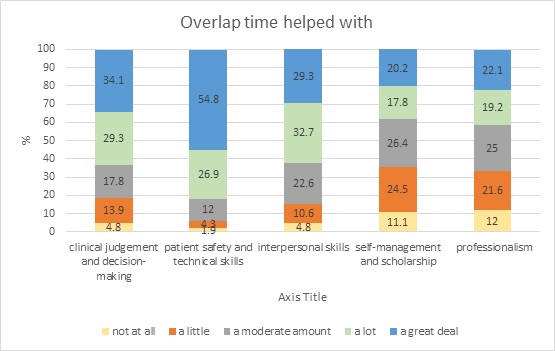


**Figure 1- Intern rating value of overlap period for achievement of core domains of practice**

Supplement: online supplemental file 1 [file bmjopen-16-3-s001.zip › bmjopen-2025-108611-20260319144200/graphic/figure1.docx]

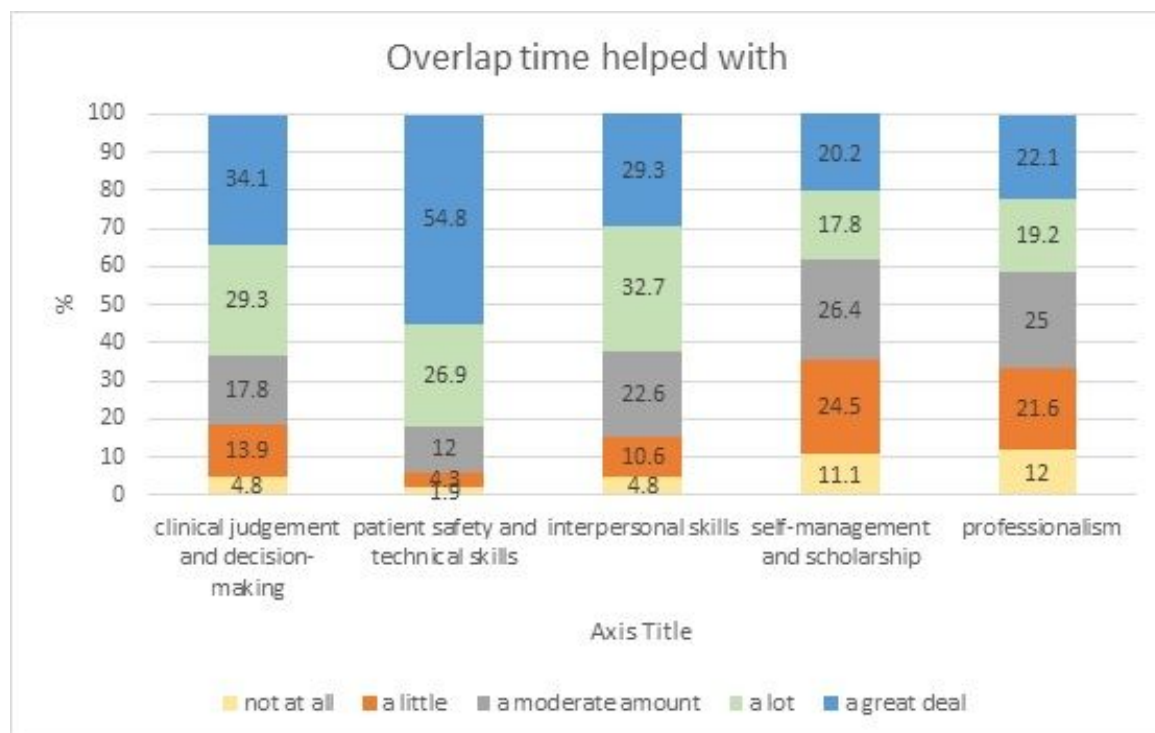

**Figure 1- Intern rating value of overlap period for achievement of core domains of practice**

Supplement: online supplemental file 1 [file bmjopen-16-3-s001.zip › bmjopen-2025-108611-20260319144200/pdf_renditions/bmjopen-2025-108611-File002.pdf]
