# Supplementary material for: Descriptive analysis of interns’ basic psychological needs, burnout and empathy in the COVID-19 pandemic in Ireland
Source: BMJ Open. 2026 Mar 30;16(3):e108611. doi: 10.1136/bmjopen-2025-108611 (PMC13052534; doi:10.1136/bmjopen-2025-108611)
Supplement: online supplemental file 1 [file bmjopen-2025-108611.RH.pdf › bmjopen-2025-108611-20260319144200/doc/bmjopen-2025-108611.RH.pdf]

PEER REVIEW HISTORY

BMJ Open publishes all reviews undertaken for accepted manuscripts. Reviewers are asked to complete a checklist review form and are provided with free text boxes to elaborate on their assessment. These free text comments are reproduced below.

ARTICLE DETAILS

Title (Provisional)

Descriptive analysis of Interns' basic psychological needs, burnout and empathy in the COVID-19 pandemic in Ireland

Authors

Patterson, Aileen; Brady, Conan; Burke, Elaine; Castello, Stefania; Courtney, Laura; F. Curley, Gerard; Donnelly, Suzanne; Hennessy , Martina; McLoughlin, Declan M.; O'Connell , Finbarr; Roberts, TE; ten Cate, Olle; Wald, Hedy; Zgaga, Lina

---

VERSION 1 - REVIEW

**Reviewer**	**1**
**Name**	**Brown, Roger**
**Affiliation**	**University of Wisconsin Madison, Research Division**
**Date**	**12-Sep-2025**
**COI**	**None**

---

1. The paper lacks a standard table 1 describing the sample.

2. No multivariable regression models were used, which would allow adjustment for potential confounders (e.g., gender, clinical setting, exposure to COVID patients).

3. The analysis is heavily univariate and bivariate, limiting the ability to disentangle independent predictors.

4. According to the author's findings, relationships in Table 2 may be partially muddled by exposure intensity since COVID-19 exposure is linked to certain outcomes (e.g., higher PA among those who routinely care for COVID patients; DP disparities by number of COVID patients). Making adjustments (e.g, partial correlations) would check if the relationships between BPNSF and burnout/empathy endure regardless of exposure. Beyond variations in pandemic exposure, correlations corrected for COVID exposure would support the idea that need satisfaction/frustration is related to burnout/empathy.

5. These results mean that COVID exposure was not neutral, it was statistically associated with at least two burnout outcomes (PA and DP). That's why I suggested partial correlations, without controlling for exposure, some of the bivariate correlations in Table 2 might partly

reflect differences in exposure intensity rather than direct associations between psychological needs and burnout/empathy.

6. With multiple comparisons in table 2, there is a risk of Type I error inflation. No correction (e.g., Bonferroni, FDR) is reported.

7. Effect sizes are not presented, which limits interpretation of the practical significance of findings.

8. Gender differences were only partially explored; more nuanced analyses could examine interaction effects.

9. Possible recall bias in reporting COVID exposures and experiences.

10. A 21% response rate raises concerns about nonresponse bias.

11. Since data were collected across a range of Irish hospitals, the authors should indicate how many hospitals. Also, how many Interns were clustered within hospitals, yet no adjustment for clustering was reported. This may underestimate standard errors.

12. The authors should document within scale missingness. How much missing occurred within each scale, and how it was handled.

13. General comment: Since I'm a statistician, I can't comment on the manuscript's qualitative section. In conclusion, the techniques and analysis are performed with competence, although their statistical sophistication is a little limited. Although the study offers valuable descriptive and correlational insights, more sophisticated multivariable analysis, multilevel modeling, and longitudinal designs should be used in future studies to support causal inference.

VERSION 1 - AUTHOR RESPONSE

Reviewer: 1
Prof. Roger Brown, University of Wisconsin Madison
Comments to the Author:
1. The paper lacks a standard table 1 describing the sample.

Thanks , Table 1 has been inserted to describe the sample.

2. No multivariable regression models were used, which would allow adjustment for potential confounders (e.g., gender, clinical setting, exposure to COVID patients).

Thank you for the comment. The quantitative data has been investigated via multivariate logistic regression for predictors of burnout. The model included gender, contact with COVID-19 patients (regularly, intermittently, rarely), testing positive for COVID-19, and relevant experiences during overlap period, specifically whether overlap period helped with interpersonal skills, self-management and scholarship, or professionalism. Final covariate included each individual subscale score for the BPNSF scale (tested) one at a time due to strong correlation between subscales. Statistical significance was defined as $p < 0.05$.

3. The analysis is heavily univariate and bivariate, limiting the ability to disentangle independent predictors.

See above, multivariate analyses have been added.

4. According to the author's findings, relationships in Table 2 may be partially muddled by exposure intensity since COVID-19 exposure is linked to certain outcomes (e.g., higher PA among those who routinely care for COVID patients; DP disparities by number of COVID patients). Making adjustments (e.g, partial correlations) would check if the relationships between BPNSF and burnout/empathy endure regardless of exposure. Beyond variations in pandemic exposure, correlations corrected for COVID exposure would support the idea that need satisfaction/frustration is related to burnout/empathy.

The relationships we checked with multivariable analyses models that adjusted for frequency of caring for COVID patients and COVID positivity. All BPNSF subscales (in separate models) were statistically significantly associated with burnout: Autonomy satisfaction (OR=0.38, p=6.12*10-6), Autonomy frustration (OR=1.67, p=0.0024), Competence satisfaction (OR=0.41, p=0.000126), Competence frustration (OR=1.42, p=0.0139), Relatedness satisfaction (OR=0.51, p=0.000226) and Relatedness frustration (OR=1.43, p=0.0394). Other covariates (namely, gender and experience during the overlap period in addition to COVID-19 positivity, frequency of dealing with COVID-19 patients) were not associated with burnout .
This has been added to results section.

5. These results mean that COVID exposure was not neutral, it was statistically associated with at least two burnout outcomes (PA and DP). That's why I suggested partial correlations, without controlling for exposure, some of the bivariate correlations in Table 2 might partly reflect differences in exposure intensity rather than direct associations between psychological needs and burnout/empathy.

Covid exposure was investigated in terms of the doctor's COVID positivity and/or frequency of dealing with Covid positive patients, as above these covariates were not associated with burnout.

6. With multiple comparisons in table 2, there is a risk of Type I error inflation. No correction (e.g., Bonferroni, FDR) is reported.

Thanks for this comment. Many variables are strongly correlated, but The Bonferroni correction assumes that all tests are independent. When variables are correlated, this assumption is violated, so the correction becomes overly conservative. As a result, it inflates the risk of Type II errors, making it too stringent for correlated data. We however note that many p-values are very small and would suggest significance even at Bonferroni correction. We have added the following to the limitations section:

"Although multiple testing corrections were considered, we retained the nominal significance threshold of P ≤ 0.05. Many of the variables examined are correlated rather

than independent, and applying simple corrections such as Bonferroni would therefore be overly conservative and increase the likelihood of Type II errors."

7. Effect sizes are not presented, which limits interpretation of the practical significance of findings.

Thank you. Effect sizes are given for multivariate models we added.

8. Gender differences were only partially explored; more nuanced analyses could examine interaction effects.

The statistical power is questionable for interaction effects, beyond the scope of the manuscript

9. Possible recall bias in reporting COVID exposures and experiences.

This was added to the limitations paragraph "There is a potential for response bias **and recall bias** as respondents self-reported on their level of burnout."

10. A 21% response rate raises concerns about nonresponse bias.

We recognise that the response rate of 21% is relatively low and therefore represents a limitation of the study. The number of responses is greater than previous large national surveys involving this cohort, however it raises the possibility of non-response bias, as individuals who chose to participate may differ from those who did not. Although the sample still provides valuable insight into national patterns, the findings should be interpreted with appropriate caution and this will be acknowledged in the limitations.

The following text on page 18 of the original manuscript has been amended to address this comment

"There is a potential for response bias ***and recall bias*** *as respondents self-reported on their level of burnout. Interns who were experiencing burnout may not have completed the survey or contrarily, may have been more likely to complete the survey due to its relevance to them. The response rate of 21% may not be representative of all interns."*

11. Since data were collected across a range of Irish hospitals, the authors should indicate how many hospitals. Also, how many Interns were clustered within hospitals, yet no adjustment for clustering was reported. This may underestimate standard errors.

Hospital-level data were intentionally not collected for this national study in order to protect the anonymity of both participating institutions and individual respondents. Because many hospitals—particularly those in smaller regions—have unique service profiles or staffing structures, collecting identifiable site information could have allowed for inadvertent re-identification of participants. To minimize this risk and ensure full confidentiality, only aggregated national-level data were obtained and analysed. This approach was approved by the National Research Ethics Committee which was convened in 2020 in response to the COVID 19

pandemic, and is consistent with their best practices for safeguarding participant anonymity in large-scale health research during this time.

12. The authors should document within scale missingness. How much missing occurred within each scale, and how it was handled.

Missing data is shown in tables 2, 4, 5

The following text has been added to the methods

“Analyses were conducted using complete-case (listwise deletion) analysis, where cases with missing data on any variable in the model were excluded.”

13. General comment: Since I'm a statistician, I can't comment on the manuscript's qualitative section. In conclusion, the techniques and analysis are performed with competence, although their statistical sophistication is a little limited. Although the study offers valuable descriptive and correlational insights, more sophisticated multivariable analysis, multilevel modeling, and longitudinal designs should be used in future studies to support causal inference.

Reviewer: 1
If you have selected ‘Yes’ above, please provide details of any competing interests.: Not applicable
